# Betweenness centrality for temporal multiplexes

**DOI:** 10.1038/s41598-021-84418-z

**Published:** 2021-03-01

**Authors:** Silvia Zaoli, Piero Mazzarisi, Fabrizio Lillo

**Affiliations:** 1 Department of Mathematics, University of Bologna, Bologna, Italy; 2 The Abdus Salam International Center for Theoretical Physics (ICTP), Trieste, Italy; 3 Scuola Normale Superiore, Pisa, Italy

**Keywords:** Complex networks, Mathematics and computing

## Abstract

Betweenness centrality quantifies the importance of a vertex for the information flow in a network. The standard betweenness centrality applies to static single-layer networks, but many real world networks are both dynamic and made of several layers. We propose a definition of betweenness centrality for temporal multiplexes. This definition accounts for the topological and temporal structure and for the duration of paths in the determination of the shortest paths. We propose an algorithm to compute the new metric using a mapping to a static graph. We apply the metric to a dataset of $\sim 20$k European flights and compare the results with those obtained with static or single-layer metrics. The differences in the airports rankings highlight the importance of considering the temporal multiplex structure and an appropriate distance metric.

## Introduction

Centrality metrics are among the most common tools used to characterise the nodes of a network. These metrics individuate the key nodes having an important role for the transmission of information through the network. In particular, betweenness centrality^1^ measures the importance of a node for the flow of information between pairs of nodes, assuming that information travels through geodesics, i.e. the shortest paths available between a pair of nodes. This metric has found applications especially for transportation, communication and infrastructural networks, where it can be used as a planning tool. For example, it can be used to evaluate traffic loads^2^ or to identify vulnerable nodes^3^. The original betweenness centrality applies to static and single-layer networks. However, many real-world networks are dynamic^4^ and multi-layer^5–7^. Both the temporal and the multi-layer structure have important effects on the network functioning. For example, multiplexity eases cooperation in social interactions^8^ and determines a non-trivial optimal condition for mobility in transportation and communication networks^9^. The temporal structure of the network critically influences dynamical processes^10^, because these processes follow time-respecting paths. Much less attention has been devoted to the investigation of the interplay of these two aspects of network structure^11^ or to methods that apply to temporal multiplexes^12^.

We argue that both the temporal and multiplex structure must be accounted for when computing centrality metrics, as they both have a comparable influence on the flow of information^13^. The temporal structure implies that information (e.g. traffic, epidemics, rumours) can only flow along time-ordered paths, and the determination of the shortest paths might depend both on time-related properties (e.g. path duration) and on the topological length. Flow across layers of a multiplex might be hindered with respect to intra-layer flow, and consequently the length of a path would depend on the presence of inter-layer jumps. A concrete example of temporal multiplexes where both aspects of the structure influence the flow are transportation networks. In transportation networks, different layers represent different transportation modes or providers (e.g. different airlines). From the temporal point of view, transportation networks have particular characteristics that determine when a path is temporally feasible. First, a non-zero time is required to travel through a link (e.g. the duration of a flight), and secondly a minimum connecting time at nodes might be needed (e.g. minimum connecting time between two flights). These characteristics have rarely been considered in previous works on temporal networks (though see^13–15^).

In the following, we consider the air transportation network as an application, but there are other applications for which the temporal multiplex approach is suited. Another transportation example is a multimodal network including trains, buses and flights, where layers represent the different transportation modes. Besides transportation networks, if we restrict to the case with instantaneous travel time and connecting time, networks of direct contacts between people represent another relevant application. In these networks, temporal links connect people that had a direct proximity contact, and contacts can take place in different contexts or communities to which people belong, represented by different layers. In the presence of a dynamical process, such as the spreading of information, both the temporal and multiplex structure play an important role. The temporal structure of contacts determines the possible paths followed by the process, and inter-layer links can represent a “friction” for the passage of information from one community to another.

To motivate why considering the temporal and multiplex structure of the network is crucial to rank nodes, let us analyse how disregarding this structure may result in wrong estimation of nodes’ importance. On a static single-layer network, the betweenness *b* of a node *i* is defined as

1 $$\begin{aligned} b(i)=\sum _{j,k}\frac{\sigma _{jk}^i}{\sigma _{jk}}, \end{aligned}$$

where $\sigma _{jk}$ is the number of shortest paths from *j* to *k* and $\sigma _{jk}^i$ is the number of such shortest paths that pass through *i*. This standard notion of betweenness centrality could be applied to temporal multiplexes circumventing their structure in two ways: (i) by aggregating the network across time and layers, and computing the metric on the resulting static single-layer network, or (ii) by computing the metric on each single layer at each single time-step and then aggregating the results^16^. These methods however discard structural information relevant for the determination of geodesics. For example, if we compute the betweenness in the single layers, all the inter-layer paths are neglected, thus underestimating the importance of nodes acting as a bridge between layers. If instead betweenness is computed on the network obtained aggregating layers, an intra-layer path and inter-layer path using *n* links are considered of the same length, although, depending on the application, the latter should be considered longer. This last procedure thus potentially overestimates the importance of bridge nodes. Time-wise, considering single time-steps does not provide meaningful information if the time scale of the information flow is the same or larger than the time scale of the network evolution (which is the case e.g. for passenger flow in transportation networks). If we instead aggregate over time, we are not able to distinguish the time-ordered paths, the only ones on which information can travel in the original temporal network. These observations call for a truly multiplex and temporal formulation of betweenness centrality.

We introduce a betweenness centrality accounting for both the temporal and the multiplex structure of the network. To this aim, we first introduce a definition of path length that combines duration, topological distance and changes of layers. Then, we propose a method to find the shortest paths that minimise this length and compute the centrality. The method is based on a mapping from the temporal multiplex to a suitable static single-layer graph. Finally, we present an empirical application of the new betweenness centrality to a dataset of flights of the European air traffic network.

Our work provides a significant addition to the previous literature in two ways. First, it considers both the multiplex and the temporal structure, differently from previous works that dealt either with single-layer temporal networks or with static multiplexes. The development of a method for this more general case requires considerable modifications of previously proposed methods. In fact, the mapping from the temporal multiplex to the static single-layer graph necessary to compute the shortest paths is specific to the characteristics of the problem. In particular, a suitable and non-trivial design of links on the static single-layer graph is required to translate each path of the temporal multiplex to a single path of the static single-layer graph with the correct length. Second, concerning the temporal structure, our method applies to networks with the following characteristics: i) non-zero link travel time; ii) minimum connecting time; iii) distance depending both on duration and topological length. (Note that the method is flexible with respect to these characteristics, it still applies to networks where i or ii are not true.) The method to find shortest paths and compute betweenness centrality depends on all these characteristics, and no previous work applies to networks of this type. Accounting for these characteristics is crucial for transportation networks, making our method more suited than previous ones for these kind of application, even when we restrict to single-layer temporal networks.

## Related work

### Temporal networks

A number of previous works has focused on paths, distances and betweenness in single-layer temporal networks. The networks considered typically have instantaneous links^17–20^, but in some cases a non-zero link travel time has been considered^14,15,21^. In^21^, the travel time is assumed to be the same for all links and equal to the discretisation interval. All these works consider paths that are time-respecting. Ref.^15^ also considers constraints on the minimum and maximum connecting time in a node (a maximum time could apply e.g. to disease spreading, to describe a finite infectious period).

Several definitions of distance have been introduced, reflecting the fact that different characteristics of paths are relevant for different application. Habiba et al.^18^ consider two types of shortest paths, those that minimise the topological length and those that minimise the duration. According to Kim and Anderson^21^ and Tang et al.^20^, the shortest path is the one arriving earliest. Bui-Xuan et al.^17^ considers three types of shortest paths: minimising topological length, minimising duration, and arriving earliest. Wu et al.^14^ considers an additional type, the ones departing latest. Himmel et al.^15^ consider 8 types of shortest paths, including all the previous ones and adding the paths that minimise the time spent travelling (duration minus total connecting time), the ones that minimise the waiting time at intermediate nodes (total connecting time), the cheapest (if a cost is associated to each link), the most likely (if a probability is associated to each link). An attempt to reconcile different definitions has been done by Tsalouchidou et al.^19^, defining the length of a path as a combination of the number of links *n* and the duration ${\mathcal {T}}$, with a relative weight $\alpha$: ${\mathcal {L}}=\alpha n+(1-\alpha ){\mathcal {T}}$. This definition summarises different aspects of a path quantifying, in a sense, its efficiency.

We remark that the choice of a distance definition depends on the application. For example, taking the shortest paths to be the one arriving the earliest could be suited in the case of information spreading on a network of contacts. In this case, in fact, it might be irrelevant how long the information travels and how many nodes it touches before arriving at its destination, while it is relevant that it arrives early. However, the same definition is not suited for a transportation network. In fact, according to this definition an itinerary arriving earlier would be always considered shorter with respect to one arriving later, regardless of the number of legs and of the travel time. In the air traffic example, a 3-legs flight itinerary departing at 8 am and arriving at 3 pm would be considered shorter than a direct flight departing at 1 pm and arriving at 3:30 pm. While arrival time is certainly not irrelevant for transportation networks, using it as the only criterion leads to unrealistic results.

Among the cited articles, only^18,21^ and^19^ discuss how to compute betweenness centrality from shortest paths on temporal networks. Tsalouchidou et al.^19^ use the definition in Eq. (1). Habiba et al.^18^ propose instead that a node should get from each shortest path passing from it a contribution proportional to the time the path dwells in the node. In^21^, the betweenness of a node in a time window $[t_i, t_j]$ is obtained summing its betweenness in each window $[t, t_j]$ with $t\ge t_i$, to account for the fact that the shortest paths could change as time proceeds. Note however that this creates an asymmetry by which paths in the late part of the time window contribute more to centrality than paths in the early part.

All articles propose a method to find the shortest paths according to their definition and to compute the betweenness (for those articles that address it). The methods are specific to the definition of distance considered, and depend on whether a non-zero link travel time is allowed. They also depend on whether the shortest paths are to be used in the computation of betweenness or not. In fact, to compute betweenness the method must return all possible shortest paths between a pair of nodes, a problem that is more complicated than finding only one shortest path per pair. Kim and Anderson^21^ propose to transpose the temporal network to a static graph where for each original node there is a ‘copy’ for each temporal step, and each copy is connected to the following one. The path arriving the earliest to node *u* starting from node *v* is then the shortest path on the static graph among those joining the copy of node *v* at the initial time step to any copy of node *u*. The shortest paths can be therefore computed on this static graph with ‘standard methods’. This procedure is specific to the definition of shortest paths as the paths arriving the earliest. Habiba et al.^18^ also propose a similar transposition to a static graph, where additionally all the copies of node *i* have an incoming link from a dummy node $i_{in}$ and an outgoing node to a dummy node $i_{out}$. The shortest paths between each pair of nodes can be computed applying Dijkstra’s algorithm between the ‘in’ and ‘out’ dummy nodes. (Note however that Dijkstra’s algorithms, unless appropriately modified, finds only one shortest path between each pair.) Tsalouchidou et al.^19^ transpose the temporal network to a weighted static graph such that the weight of a path is equal to its length (according to the definition they propose) in the temporal network. Shortest paths can be found, again, using Dijkstra’s algorithm with an ‘in’ dummy node a source.

### Multiplexes

Boccaletti et al.^5^ introduce the notions of paths and distances for multiplex networks. They note that, when computing the length of a path made of intra- and inter-layer links, the latters might have a larger weight.

Solé-Ribalta et al.^22^ propose a definition of betweenness centrality for (static) multiplexes and a method to compute it. They assume that the length of a path is given by the number of intra-layer links. They define the betweenness centrality of a node *v* as $b(v)=\sum _l b(v_l)=\sum _l \sum _{s,t \ne v}\frac{\sigma _{s,t}(v_l)}{\sigma _{s,t}}$, where $\sigma _{s,t}$ is the number of shortest paths connecting node *s* on any layer to node *t* on any layer, and $\sigma _{s,t}(v_l)$ is the number of such paths passing from the copy of node *v* on layer *l*. Note that this definition gives more centrality to nodes traversed by inter-layer shortest paths, as these paths contribute to the centrality of two copies of the node, which are then summed. They compute the shortest paths using Breadth Fist Search or Dijkstra’s algorithm, and then betwenness is computed through an algorithm inspired by the one by Brandes^23^.

### Temporal multiplexes

The literature on temporal multiplexes lacks a specific definition of betweenness centrality as well as a method to compute it. In our previous work^13^, we introduced a generalisation of Katz centrality for temporal multiplexes, accounting for non-zero link travel time. However, such generalisation does not address the problem of distances and shortest paths in temporal multiplex. The present work fills the gap.

## A method to compute temporal multiplex betweenness

In this section we present the method we propose to compute betweenness centrality on a temporal multiplex. First, we define feasible paths on a temporal multiplex and introduce a notion of distance. Then, we propose a method to find the path minimising these distances and compute betweenness centrality.

### Paths and distance on temporal multiplexes

Consider a temporal multiplex $G=(V, E, I)$ where *V* is a set of *N* nodes, identical on each of the *M* layers, *E* is a set of intra-layer links, and *I* is a set of inter-layer links connecting a node to its copies on the other layers. For simplicity, we consider the case in which all copies of each node are connected at all times, but our method can be easily adapted to different choices. In the air traffic application nodes represent airports and intra-layer links represent flights, while layers represent different airlines. Each link $e\in E$ is characterized by a time of appearance and a time of disappearance, their difference representing the time it takes to travel through that link.

A valid path in this temporal multiplex is a sequence of edges $\{e_1, \dots , e_n\}\in E$ such that if $e_i$ is incident to node $j\in V$ on layer $\lambda$ and disappears (‘arrives’) at time *t*, then $e_{i+1}$ leaves from node *j* on any layer and appears (‘departs’) at time $t'\ge t+\delta t$, where $\delta t$ is a minimum connecting time (as also introduced in^15^).

To define a betweenness centrality for temporal multiplexes, we need to define a notion of distance to individuate the shortest paths. For temporal networks, several definitions of distance exist^14,15,17,18,21^, ranging from pure topological distances considering the number of links, to purely temporal ones considering the path duration or time of arrival or other time-related properties. We propose a definition combining duration, topological distance and changes of layers. This definition generalises the definition of Ref.^19^, which applies to single-layer temporal networks, to the multiplex case. The length of a path ${\mathcal {L}}$ is a combination of the temporal duration ${{\mathcal {T}}}$ (from the departure of the first link to the arrival of the last one) and the topological length, determined by the number of intra-layer link used, *n* and the number of inter-layer link used, *m*:

2 $$\begin{aligned} {\mathcal {L}}=\alpha (n+\varepsilon m) + (1-\alpha ) {{{\mathcal {T}}}}, \end{aligned}$$

where $\alpha \in [0, 1]$ and $\varepsilon \in [0, \infty )$. The shortest path on the temporal multiplex *G* is the one miminimizing ${\mathcal {L}}$.

The parameter $\alpha$ tunes the relative weight of the topological length and the duration: when $\alpha =0$, ${\mathcal {L}}$ is the duration, while when $\alpha =1$ it is the topological length. The parameter $\varepsilon$ determines the cost of each inter-layer link. When $\varepsilon =0$, a path using *m* inter-layer links and *n* intra-layer ones has the same topological length as an intra-layer path using *n* links. When $\varepsilon =1$ the topological cost of an inter-layer link is the same as that of an intra-layer one. The value of the parameter $\varepsilon$ measures the propensity of information to jump between layers, or the associated cost, therefore its most fitting value depends on the application. For example, for the application to the air transportation network that we show below, a value $\varepsilon >0$ is realistic because flight itineraries with inter-airline connections are risky from the passengers’ point of view and therefore intra-airline paths are preferred.

### Finding the shortest paths to compute betweenness centrality

**Figure 1:**
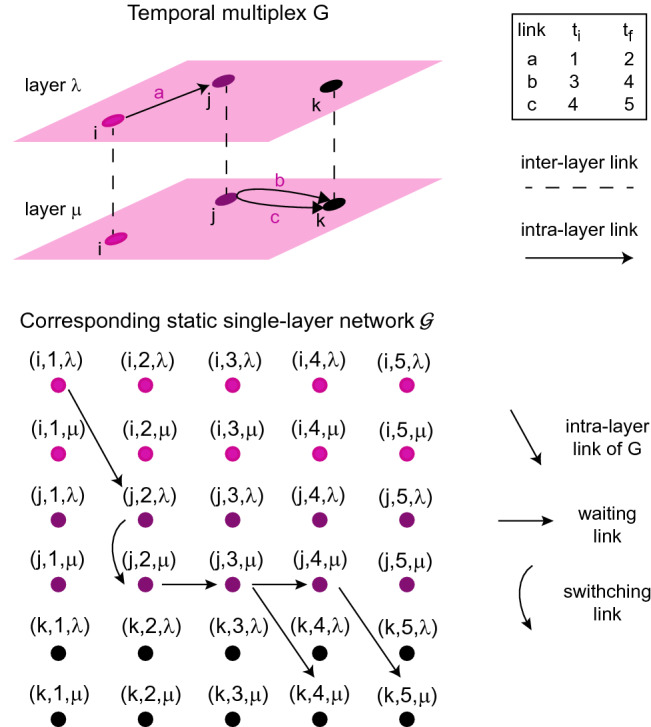
Example of conversion of a temporal multiplex *G* (above) into the corresponding static single-layer network ${\mathcal {G}}$ (below). *G* has $M=2$ layers and $N=3$ nodes, links *a*, *b* and *c* have the temporal structure ($T=5$) indicated on the side ($t_i$ is the time-step during which the link appears and $t_f$ the one during which it disappears). In ${\mathcal {G}}$ each of the three nodes has 10 copies, one per layer and per time-step of the temporal discretization.

Once the shortest paths between all pairs of the *N* nodes in *V* are found according to the proposed definition of path length, betweenness can be computed according to Eq. (1). Note that the shortest path between *i* and $j\in V$ is the shortest among all the paths joining the two nodes on any two layers and at any time. In order to find all such shortest paths, we propose the following algorithm, that adapts the procedures used in single-layer temporal networks in^18,19,21^:

1. the temporal multiplex *G* is converted into a static single-layer network ${\mathcal {G}}=({\mathcal {V}}, {\mathcal {E}})$ (see Fig. 1), such that the paths of ${\mathcal {G}}$ are all the feasible temporal paths of *G* and each path weight (sum of links’ weights) is equal to the path length ${\mathcal {L}}$ in *G*;
2. the shortest paths between all pairs of nodes of ${\mathcal {G}}$ are found using Dijkstra’s algorithm^24^;
3. among all the shortest paths found, we select only those that are shortest also on the original temporal multiplex *G*.

Let us detail better each step.

To convert the temporal multiplex into a static single-layer network we discretize time in windows of length $\Delta t$, obtaining *T* time-steps (see SI for an analysis of the effects of time discretization). For each vertex $i\in V$, in ${\mathcal {V}}$ we have a ‘copy’ of *i* for each time-step and each layer. That is, each vertex *i* of the temporal multiplex corresponds to a set of $N\times T \times M$ vertices $\nu =(i,t,\lambda )$ with $t=1, \ldots , T$ and $\lambda =1, \ldots , M$.

Then, we add weighted links in the static network such that each path in this network is a feasible temporal path on the temporal multiplex, with weight equal to the path length on the temporal multiplex. First, all intra-layer links $e\in E$ are translated into links $\epsilon \in {\mathcal {E}}$ of the static network. Specifically, a link joining vertex *i* to vertex *j* on layer $\lambda$, lasting from $t_1$ to $t_2$, in the static network becomes a link from $\nu =(i,t_1,\lambda )$ to $\mu =(j,t_2,\lambda )$ (Fig. 1). This link is given a weight $\alpha +(1-\alpha )(t_2-t_1)$, which accounts for its topological length and for its duration. Additionally in the static network we have ‘switching’ links and ‘waiting’ links (Fig. 1). Switching links allow paths to switch layer after using an intra-layer link. They are directed links between two copies of *i* on different layers, $(i,t,\lambda )$ and $(i,t,\eta )$. Switching links are present at each time *t* at which an intra-layer link ends in vertex *i* on layer $\lambda$, they are directed to all other layers and weight $\alpha \varepsilon$ . Waiting links allow a path to wait in one node between the usage of two intra-layer links. Given a vertex $\nu =(i, t_1,\lambda )$ with an incident link (either intra- or inter-layer), a waiting link joins it to the the the vertex $\mu =(i, t_2, \lambda )$, where $t_2$ is the earliest successive time-step at which a link starts from *i* on layer $\lambda$. This waiting link allows a path that arrived in *i* at time $t_1$ (on any layer) to wait until time $t_2$ to take the earliest next link on layer $\lambda$. Additionally, a path might not use the earliest next link, but a successive one. To allow this, every time that a link starts from *i* on layer $\lambda$, a waiting link is present to the earliest successive time-step at which another link starts from *i* on the same layer, so that the path can still wait and use a successive link. A waiting link joining $(i,t_1, \lambda )$ to $(i,t_2,\lambda )$ is weighted $(1-\alpha )(t_2-t_1)$, according to its duration.

In summary, a path on the static graph can use an intra-layer link, then either wait on the same layer for a further link or jump to another layer and wait there. Waiting links can be used one after the other (in case the path does not use the earliest next link but a further one). The weight of the path is given by the sum of the weight of its links.

As noted in ref.^15^, it is possible to account for a minimum connecting time of $\delta t$ needed between one intra-layer link and the other by simply assigning to each original link $e\in E$ an extra duration of $\delta t$. This will increase the weight of every path by $(1-\alpha )\delta t$, because the last link will arrive at a time $\delta t$ later and thus increase the total duration, but it will not affect the ranking of their length. Every path on ${\mathcal {G}}$ corresponds to a time-ordered path on *G*, and its weight corresponds to the length ${\mathcal {L}}$ of the original path. It is however possible that one path on *G* has more than one corresponding paths on ${\mathcal {G}}$, with the same weight. These ‘cloned’ paths are a side-effect of our path-counting method, and they coincide except for the fact that they change layer at a different time-step or, in the case $\alpha =1$, for an additional ‘free’ wait at the origin or destination node. In the SI we detail when these ‘cloned’ paths are present and how to exclude them from the counting.

Dijkstra’s algorithm is applied to each of the *NTM* nodes of ${\mathcal {V}}$ to find the shortest paths from that node to each of the others, with a run-time for each node scaling as $O((NTM+L(M+2))\log (NTM))$, with *L* the number of links in *G*. Assuming the network is sparse at each time step ($L \propto NT$), the complexity of this phase of the algorithm is $O((NTM)^2 \log (NTM))$. We thus obtain a set of shortest paths of ${\mathcal {G}}$ that we can map to paths of *G*, by recalling that all nodes $(v,t,\lambda )\in {\mathcal {V}}$ , $\forall t, \lambda$ correspond to node $v\in V$. Not all these paths are geodesics in *G*: for example if there are two links between *u* and *v* appearing at different time steps and such that the first has a smaller duration than the second, both would be shortest paths of ${\mathcal {G}}$ but only the first would be a geodesic in *G*. Therefore we select only those that are geodesics in *G* by noting that, of all the shortest paths found between any copy of *v* and any copy of *u* in ${\mathcal {V}}$, only the shortest ones are geodesics between *v* and *u* in *G*. We then use them to compute betweenness centrality. Note that the efficient recursive algorithm by Brandes^23^ to compute betweenness given the shortest paths cannot be used in this case because the condition that subpaths of shortest paths are shortest paths themselves is not satisfied for a temporal network.

The computational complexity of the algorithm is typically dominated by the application of Dijkstra’s algorithm (see SI for details), and is therefore $O((NTM)^2 \log (NTM))$.

## Numerical results: application to the European air traffic network

We illustrate the critical importance of accounting for the temporal multiplex structure by comparing the nodes’ ranking obtained with the proposed betweenness centrality for temporal multiplex and with previously available methods, on the European air traffic network.

### Dataset and network construction

The dataset contains all flights passing through ECAC (European Civil Aviation Conference) airspace on September 1st, 2017. Countries in the enlarged ECAC space are: Iceland (BI), Kosovo (BK), Belgium (EB), Germany-civil (ED), Estonia (EE), Finland (EF), UK (EG), Netherlands (EH), Ireland (EI), Denmark (EK), Luxembourg (EL), Norway (EN), Poland (EP), Sweden (ES), Germany-military (ET), Latvia (EV), Lithuania (EY), Albania (LA), Bulgaria (LB), Cyprus (LC), Croatia (LD), Spain (LE), France (LF), Greece (LG), Hungary (LH), Italy (LI), Slovenia (LJ), Czech Republic (LK), Malta (LM), Monaco (LN), Austria (LO), Portugal (LP), Bosnia-Herzegovina (LQ), Romania (LR), Switzerland (LS), Turkey (LT), Moldova (LU), Macedonia (LW), Gibraltar (LX), Serbia-Montenegro (LY), Slovakia (LZ), Armenia (UD), Georgia (UG), Ukraine (UK). We selected scheduled passenger flights (excluding e.g. charter, cargo) departing after 00:00 AM and either departing from or landing at an ECAC airport (and not only passing through the airspace). For each flight, we consider the following information: scheduled departure time, scheduled landing time, airline.

The network based on this dataset has $N=435$ nodes/airports and $M=32$ layers corresponding to single airlines or alliances. For the analysis we selected only a subset of all the airlines present in the dataset. In particular, we considered only the airlines having both a number of flights and of destinations above the average, in order to avoid having a large number of layers with few links. Note that the dataset includes a total of 183 airlines, many of which are extra-EU airlines with few flights and destinations within the ECAC space. With this selection, we retain 19648 flights. Airlines with few flights but that are part of an alliance are still retained in the analysis, because all airlines part of the same alliance form a single layer. This choice reflects the fact that connections within airlines of the same alliance are as favoured as connections within a single airline, therefore no additional ’cost’ needs to be considered. We build ${\mathcal {G}}$ with $\Delta t=15$ min (see SI for justification), corresponding to $T=116$ and a minimum connecting time $\delta t=30$ min.

We computed betweenness centrality for four different values of $\alpha$: 0, 4/5,12/13, 1. The meaning of the value of $\alpha$ can be understood as follows: when $\alpha =(1-\alpha )K$, the use of an additional link (flight) weights as much as an additional wait of *K* time-steps (on top of the duration of the link). For example, if we deem that from the passenger point of view an itinerary using $n+1$ flights has the same distance ${\mathcal {L}}$ as one using *n* flights but lasting 3h more, we would have $K=3\times \frac{60}{\Delta t}=12$ time-steps, therefore $\alpha =12/13$. We also consider four different values of $\varepsilon$: 0,0.5, 1, $\infty$. The last value corresponds to forbidding inter-layer paths, as they have infinite cost, and is simply obtained by not putting any inter-layer link in the network.

### Comparison with static betweenness on aggregated network

The first comparison we perform aims to show that considering the temporal and multiplex structure of the network yields significantly different results with respect to considering an aggregated network. With this aim, we compare the results obtained with the proposed betweenness metric with those obtained with the standard betweenness $b_{stat}$ applied on the network aggregated across layers and time-steps. We consider two ways of aggregating: (i) in the aggregated network there is a link between *i* and *j* if there is at least one temporal link among them, in any layer; (ii) for each different temporal link between *i* and *j* in *G*, there is one link in the static network (which is, therefore, a multi-link network). Note that using the proposed betweenness with $\varepsilon =0$ is equivalent to aggregating only across layers (and not across time-steps) according to method (ii).

To compare results, we consider two aspects: (1) the similarity of the rankings for the airports having non-zero betweenness according to at least one of the two compared metrics, measured by the Kendall rank correlation coefficient $\tau$; (2) the similarity between the sets of airports having zero betweenness according to both metrics, measured by their Jaccard index *J*. The coefficient $\tau$ takes values in $[-1,1]$, with 1 corresponding to two identical sequences and $-1$ to two sequences that are one the inverse of the other. The index *J* is computed as the quotient between the number of elements in the intersection and the number of elements in the union of the two sets and takes value in [0, 1], with 1 corresponding to identical sets. We remark that when $\alpha =0$ (i.e. only path duration counts), we only compare the case in which inter-layer paths are allowed ($\varepsilon <\infty )$ to the case in which they are not ($\varepsilon =\infty )$, because the value of $\varepsilon$ has no effect.

**Figure 2:**
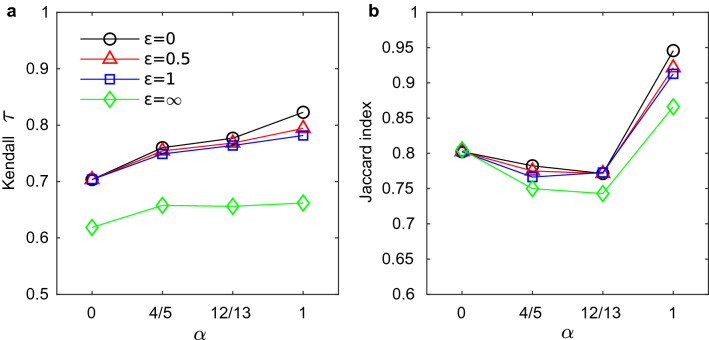
Comparison between the results obtained with the proposed betweenness centrality and with static betweenness centrality computed on the aggregated network obtained with method (i), for different values of the parameters $\alpha$ and $\varepsilon$. (**a**) Correlation between the rankings; (**b**) Jaccard index between the sets of airports with zero-betweenness according to both metrics.

The rankings obtained with the proposed betweenness metrics are always quite different from those obtained with the standard betweenness, in fact $\tau$ ranges roughly from 0.6 to 0.8 (Figs. 2a and S1a). As expected, the similarity of the rankings increases as importance of the duration decreases ($\alpha$ approaches 1) and as the weight of an inter-layer jump decreases ($\varepsilon$ approaches 0). This behaviour is expected because the single-layer betweenness does not account for path duration and does not distinguish layers.

**Figure 3:**
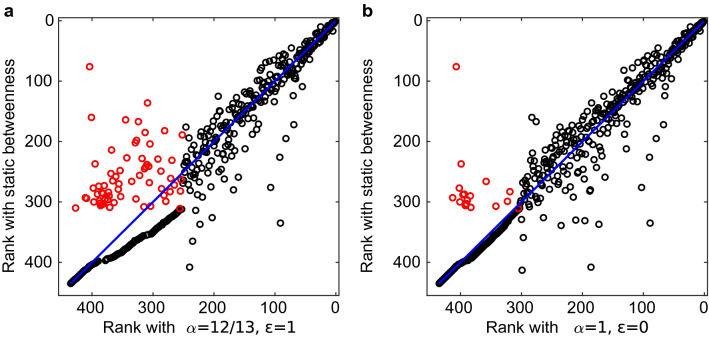
Comparison between the ranking according to the static betweenness on the aggregated network (method (i)) and the betweenness proposed here, computed with $\varepsilon =1$ and $\alpha =12/13$ (panel **a**) and $\alpha =1$ (panel **b**). Each dot represents an airport, red dots are airports having $b_{stat}>0$ but $b=0$. The blue line is 1:1.

The comparison of the sets of airports having zero betweenness according to both metrics (Figs. 2b and S1b) highlights the effects of accounting for duration. In fact, when duration is not considered ($\alpha =1$) these sets are similar, with $J\sim 0.95$, while when duration is considered they are more different, with $J\sim 0.8$ for all values of $\alpha <1$. The differences in the sets are due to a number of airports having positive betweenness on the aggregated network but null betweenness with the proposed metric. When duration is considered, there are 30 to 74 airports in this situation (Fig. 3a), while many less are found when path duration is ignored (see Fig. 3b). This situation arises because paths passing from these airports that are geodesics in the aggregated network are either not temporally ordered or not minimizing the distance ${\mathcal {L}}$ due to their duration. For the same reason some airports lose rank when the temporal multiplex structure of the network is considered. These results confirm the importance of considering the temporal and multiplex structure of the network to correctly identify the geodesics and rank nodes.

### Comparison with sum of temporal betweenness on single layers

The second comparison we perform aims to show that, even when inter-layer jumps are forbidden ($\varepsilon =\infty$), accounting for the multiplex structure as proposed produces different results with respect to summing the temporal betweenness centrality obtained on each single layer. The two procedures differ because when we sum the centralities of single layers the betweenness of node *k* is computed as $b(k)=\sum _{i,j} \sum _\lambda \frac{\sigma ^k_{i,j,\lambda }}{\sigma _{i,j,\lambda }}$, where $\sigma _{ij,\lambda }$ is the number of shortest paths between *i* and *j* on layer $\lambda$, while when we set $\varepsilon =\infty$ it is computed as $b(k)=\sum _{i,j} \frac{\sum _\lambda \sigma ^k_{i,j,\lambda }}{ \sum _\lambda \sigma _{i,j,\lambda }}$. Additionally, not all the shortest paths identified on a single layer might be shortest paths when the entire multiplex is considered, therefore the two procedures count different shortest paths. When applying the two methods to the airport network, we obtain in fact two different rankings, with $\tau =0.73$ (for $\alpha =12/13$). The two rankings, compared graphically in Supplementary Fig. S2, differ already in the highest positions. The Jaccard index is 0.89. These results show that even when inter-layer walks are prohibited it is important to take in consideration the multiplex structure.

### Sensitivity analysis

Finally, we compared the rankings obtained with the proposed betweenness for different values of $\alpha$ and $\varepsilon$ to assess the sensitivity of the results to the two parameters.

Concerning $\alpha$, when both the duration and the topological length are considered (i.e. $\alpha \in (0,1)$), we find that the ranking is quite stable when $\alpha$ varies within a meaningful intermediate range (4/5 to 12/13). In fact, $\tau \sim 0.93$ between the rankings with $\alpha =4/5$ and 12/13 for all values of $\varepsilon$ and also the sets of airports having zero betweenness are very similar ($J \ge 0.95$). When instead we compare rankings obtained with intermediate values of $\alpha$ with those obtained with the extreme values of $\alpha$, which correspond to considering only the duration or only the topological length, we observe a larger differences. For example, the rankings obtained with $\alpha =0$ and $\alpha =4/5$ (for $\varepsilon =0$) have $J= 0.82$ and $\tau = 0.86$, and those obtained with $\alpha =12/13$ and $\alpha =1$ (for $\varepsilon =0$) have $J=0.82$, $\tau = 0.88$. We therefore conclude that, to set the parameter $\alpha$, it is important to determine if for the considered application the length of a path is determined by duration, topological length or both. In case it is determined by both, however, it is sufficient to have a rough estimate of the relative importance of duration and topological length to set $\alpha$, as the ranking is robust to variations of the parameter.

**Figure 4:**
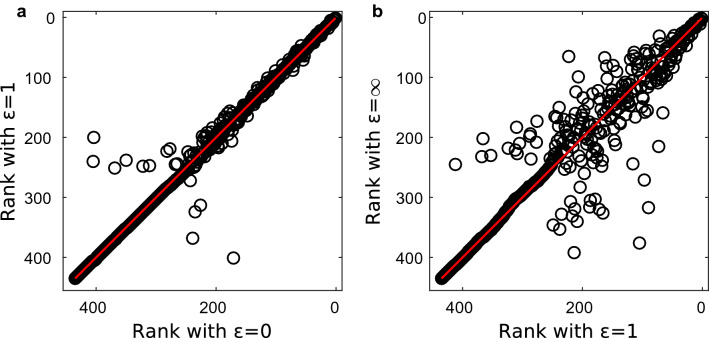
(**a**) Comparison between the rankings with $\varepsilon =0$ and $\varepsilon =1$ (for $\alpha =12/13$); (**b**) Comparison between the rankings with $\varepsilon =1$ and $\varepsilon =\infty$ (for $\alpha =12/13$) Each dot represents an airport. The red line is the 1:1 line.

Concerning $\varepsilon$, the airports’ ranking remains very similar when the value of $\varepsilon$ varies between 0 and 1. In fact, for all combinations of the values $\varepsilon =0,0.5, 1$, the rankings obtained (with $\alpha =4/5,12/13$) have $\tau \ge 0.93$ (see Fig. 4a). Larger differences are found when we compare a ranking obtained forbidding inter-layer walks ($\varepsilon =\infty$) with one obtained with finite $\varepsilon$. For example, the rankings with $\varepsilon =1$ and $\varepsilon =\infty$ have $\tau \sim 0.7$ (for $\alpha =4/5,12/13$), see also Fig. 4b. In summary, to set the parameter $\varepsilon$ it is important to determine if inter-layer path are allowed or not, but if they are allowed the specific value of $\varepsilon$ does not affect strongly the ranking. The latter observation suggests that, for a given origin-destination pair, there is rarely the choice between an inter-layer and an intra-layer walk with similar length, such that changing the cost of an inter-layer jump between 0 and 1 can make one more convenient then the other. In other words, if with $\varepsilon =0$ an inter-layer walk is the shortest between *i* and *j*, probably there is no intra-layer walk between them or it is very lengthy, therefore the inter-layer one will remain the shortest when $\varepsilon$ increases.

## Conclusions

We proposed a method to compute betweenness centrality on a temporal multiplex, based on a definition of distance that combines information on the topological distance, the path duration and the number of changes of layer. Our definition of distance includes two parameters, $\alpha$ and $\varepsilon$, which have a clear interpretation related to the relative importance of duration with respect to number of links used and to the level of hindrance that an inter-layer link poses. Their values can be chosen to fit the specific application. Our sensitivity analysis showed that, when setting the parameters values, the important choices are whether to consider both duration and topological length or only one of the two, and whether to allow inter-layer walks. Given these decisions, the result are robust to variations of the parameters.

We presented a method to find the shortest paths according to such definition of distance by converting the temporal multiplex to an appropriate static single-layer network. The paths found by this method are time-ordered, account for the potentially non-zero time required to travel through one link and for a minimum connecting time between links. The algorithm we propose is feasible on networks with $N\times M \times T \sim 10^6$, as proved by application to the European air traffic network where this condition holds.

The empirical application shows that both the temporal and multiplex aspects affect strongly the node ranking, as they both have an influence on the flow of information, therefore it is crucial to consider both if we want to estimate the importance of a node. In fact, by comparing our new metric to previous ones, i.e. static betweenness applied the aggregated network and temporal single-layer betweenness applied to each layer separately, on the network of European air transport we prove that accounting for the temporal multiplex structure of the network is crucial for a genuine assessment of the centrality ranking.

## Supplementary Information

Supplementary Information.

## Data Availability

The dataset we used for the analysis is owned by EUROCONTROL (http://www.eurocontrol.int), the European public institution that coordinates and plans air traffic control for all of Europe. Data were obtained as part of the SESAR Joint Undertaking WP-E research project DOMINO “ Novel tools to evaluate ATM systems coupling under future deployment scenarios” after competing in a public call issued by SESAR Joint Undertaking. Data can be accessed by asking permission to the legitimate owner (EUROCONTROL). The owners reserve the right to grant/deny access to data.
