# Supplementary material for: Betweenness centrality for temporal multiplexes
Source: Sci Rep. 2021 Mar 1;11:4919. doi: 10.1038/s41598-021-84418-z (PMC7921680; doi:10.1038/s41598-021-84418-z)
Supplement: Supplementary file 1 — Supplementary Information. [file 41598_2021_84418_MOESM1_ESM.pdf]

Supplementary information
Betweenness centrality for temporal multiplexes

Silvia Zaoli[1,2,*], Piero Mazzarisi[1,3], and Fabrizio Lillo[1]

[1]Department of Mathematics, University of Bologna, Bologna, Italy
[2]The Abdus Salam International Center for Theoretical Physics (ICTP), Trieste, Italy
[3]Scuola Normale Superiore, Pisa, Italy
[*]To whom correspondence may be addressed. E-mail: szaoli@ictp.it

December 17, 2020

Supplementary Figures

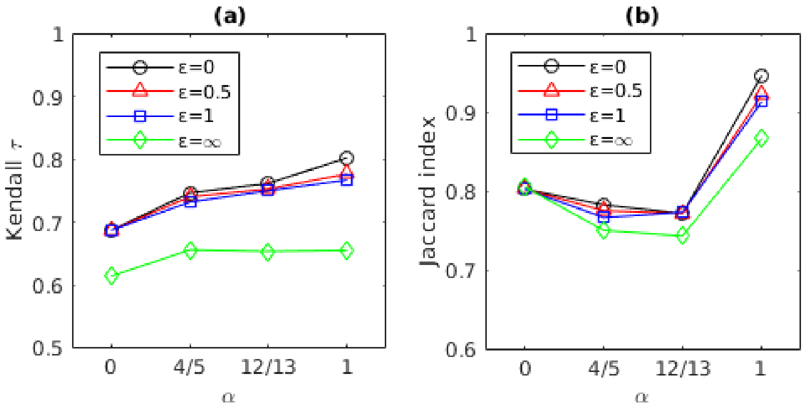

Figure S1: (a) Correlation between the ranking obtained with the proposed betweenness centrality and with static betweenness centrality computed on the aggregated network obtained with method (ii) (see main text); (b) Jaccard index between the sets of airports with zero-betweenness according to the proposed betweenness centrality and to static betweenness centrality computed on the aggregated network obtained with method (ii) (see main text), for different values of the parameters α and ε. The index J is computed as the quotient between the number of elements in the intersection and the number of elements in the union of the two sets.

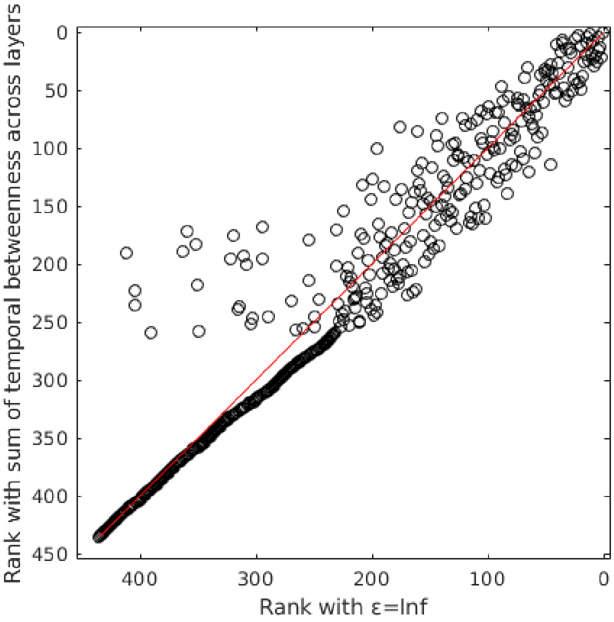

Figure S2: Comparison between the ranking according to the sum of temporal betweenness computed on each single layer ($b(i) = \sum_{\lambda=1}^{32} b_\lambda(i)$ with $b_\lambda(i)$ temporal betweenness of node i on layer λ) and the betweenness proposed here, computed with $\alpha = 12/13$ and $\varepsilon = \infty$. Each dot represents an airport. The red line is the 1:1 line.

Effect of the time discretization

To transform the temporal multiplex G into a static single-layer network $\mathcal{G}$ we need to discretize time in windows of a length Δt. This is a common approach to treat temporal networks [2–7], however the length of the temporal window must be chosen carefully so that it does not affect too much the results. Clearly, if the time window is too large with respect to the typical interval between the disappearance of an incoming link to a node and the appearance of an outgoing link from the same node, the real temporal order of links will in some cases not be respected in the static network. Taking the example of the air traffic network, suppose that we take a time window of 30 minutes. Suppose flight A lands at an airport at time t, and flight B departs from the same airport at time $t - 15min$. Then, if in the discretization the two times fall in the same window there will be a paths that takes flight A and the flight B in succession, although this is not possible in reality. If we choose $\Delta t < 15min$, instead, this path will not be possible on $\mathcal{G}$. In this example we neglected connecting time, but if we add a minimum connecting time of 30 minutes, obtained by adding 30 minutes to the duration of all flights, with a time window of 30 minutes we can have itineraries with down to no connecting time, while with a time window of 15 minutes we can have down to 15 minutes of connecting time (instead of the desired 30). The smaller Δt is, the more precisely $\mathcal{G}$ corresponds to the original temporal network and respects the imposed connecting time, if present. However, diminishing Δt increases the number of nodes in $\mathcal{G}$, and therefore the time required to run the algorithm. Therefore, choosing Δt is a trade-off between precision of the description and run time.

In figure S3 we compare the betweenness centrality obtained in the application to the ECAC air transport network (see main text for detail on the dataset) with different values of Δt of 5, 10, 15 and 30 minutes. The airports on the x-axis are ordered according to their centrality with Δt= 5 min,

the y-axis is in log-scale to enhance the differences. We observe that the difference in betweenness centrality are not very large between the different time windows, although they become larger for the less central airports. In particular, note in the left end of the plot a small number of points for which centrality is zero for smaller values of Δt (points not appearing in the log-scale plot) but not for larger ones. The obtained rankings are very similar for the most central airports, and differ slightly for the less central ones. The Kendall correlation coefficients of the obtained ranking are 0.97 for the ranking obtained with 5 and 10 minutes, 0.96 for the ranking obtained with 5 and 15 minutes, 0.93 for the ranking obtained with 5 and 30 minutes.
For the results shown in the main text we used a time window of 15 minutes together with a minimum connecting time of 30 minutes, meaning that in the worst case scenario we consider an itinerary with only 15 minutes of real connecting time. As mentioned above, this choice produces a ranking that is very similar to the one obtained with the finer discretization in 5 minutes windows.

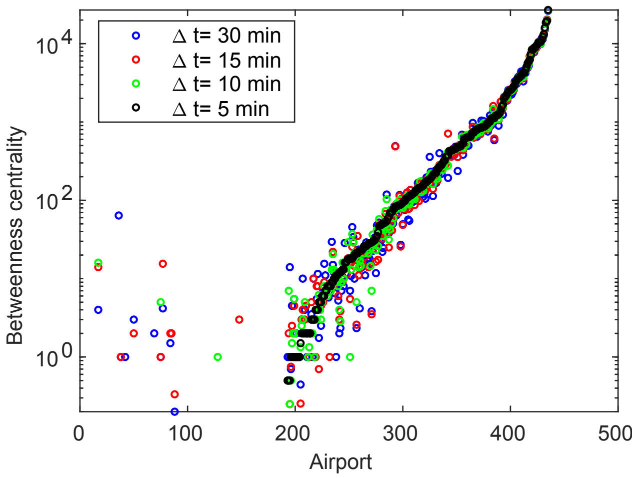

Figure S3: Comparison between the betweenness centrality obtained with different values of Δt of 5, 10, 15 and 30 minutes. The airports on the x-axis are ordered according to their centrality with Δt= 5 min, the y-axis is in log-scale to enhance the differences. Results were obtained with $\alpha = 12/13$, $\varepsilon = 0$.

Excluding 'cloned' paths from the counting

Every path on the static single-layer network $\mathcal{G}$ described in the main text corresponds to a time-ordered path on the temporal multiplex G, and its weight corresponds to the length $\mathcal{L}$ of the original path. It is however possible that one path on G has more than one corresponding paths on $\mathcal{G}$, with the same weight. This happens in two cases:
(i) For inter-layer paths when, between the time-step at which the path arrives in a node v on layer λ and the time-step at which it leaves v from layer μ, more than one inter-layer link are available to jump between layers. In fact, in this case alternative paths that correspond in everything but the time-step at which they change layer are possible on $\mathcal{G}$. Only one of these alternative paths should be counted, as they all correspond to the same path on G. This is obtained by only finding one shortest path for each pair of nodes in $\mathcal{V}$, instead of all the possible ones (when running Dijkstra's algorithm). Note that in this way we can still find several shortest paths between each pair of nodes of V. Actual shortest paths are neglected with this procedure only if there are two paths of the same length between (v, t_1, λ) and (u, t_2, μ) that actually correspond to two different paths in G. However this seems very improbable in trasportation networks for a sufficiently fine time-discretization, as it

would mean that two itineraries leave at the same time-step on the same layer to arrive at the same time-step on the same other layer;

(ii) When $\alpha = 1$, i.e. only the topological length of the path is considered. In this case, given a shortest path from i to j, a second path obtained waiting an additional time in i before the beginning or in j at the end has the same length. Therefore, for each shortest path between i and j in G several 'cloned' ones are found in $\mathcal{G}$ that differ by the waiting times in i and j. This problem can be fixed by eliminating, at the beginning and at the end of each shortest path found, the 'excess' copies of node i and node j and then removing repeated paths in the shortest paths list.

Note that (i) applies to all value of the parameters, while (ii) only to the case $\alpha = 1$. Another case to treat with care is the case in which changes of layer are free, i.e. $\varepsilon = 0$. In this case, given a shortest path from i to j, a second path that coincides with the first except for some additional changes of layer would weight the same. For example, the two paths $(i, t, \lambda) \to (j, t', \lambda)$ and $(i, t, \lambda) \to (j, t', \lambda) \to (j, t', \eta)$ are counted as two shortest paths of equal length. The solution not to have these cloned paths is simply to build $\mathcal{G}$ without copies of each node for each layer, since when $\varepsilon = 0$ the multi-layer structure has no effect on the path length.

Finally, some previous works dealing with shortest paths in temporal networks [3, 6] add to $\mathcal{G}$ dummy nodes, e.g. one outgoing dummy node i_{out} and one incoming dummy node i_{in} for each $i \in V$, such that i_{out} has an outgoing link to all copies of i and i_{in} has an incoming link from all copies of i. The weight of all links from and to dummy nodes is zero. The advantage of having dummy nodes is that one only needs to find the shortest paths between the $N \times N$ pairs of dummy nodes instead of the $NTM \times NTM$ pairs. However, with this choice it is not possible anymore to find all shortest paths between a pair i, j without counting also the cloned paths mentioned above. In fact, if we only find one shortest path for each pair of dummy nodes, we neglect potential other paths of the same length that are genuinely different paths in G. On the other hand, if we find all shortest paths between a pair (using a modified version of Dijkstra's algorithm), these will include the cloned paths of (i).

Algorithm complexity

To compute the complexity of the algorithm for a temporal multiplex with N nodes, M layers, T time steps and L links, let us divide it in three parts.

- In the first, Dijkstra's algorithm is applied to each of the $N \times M \times T$ nodes of the static graph, which has $L \times (M + 2)$ links. L is the number of original links in the temporal multiplex, which are mapped to $L \times (M + 2)$ links in the static graph. This part takes $O(NTM(NTM + L(M + 2)) \log(NTM))$. If the network is sparse at each time-step, that is, if $L \sim NT$, we get $O((NTM)^2 \log(NTM))$;

- In the second part, we select among all the shortest paths found between each pair of nodes of the static graph the ones that are shortest paths between each pair of nodes of the temporal multiplex. To do this, we have to cycle over the N^2 pairs of nodes of the temporal multiplex, and within the cycle perform operations on the shortest paths of the static graphs between copies of that pair. These operations take a time proportional to the number of such shortest paths. Given that in the static graph we find at most one shortest path for each pair of nodes, the number of shortest paths of the static graph between copies of u and copies of v is given by how many pairs of copies of u and v are connected. In the worst case scenario, where all copies of u are connected to all copies of v, #shortest paths $= O((NTM)^2)$. This case is however unrealistic: for our air traffic application it would mean that from airport v at every time step and from every layer there is an itinerary to airport u arriving at every possible time step on every possible layer. A more realistic estimate is that between two nodes v and u there are a

few possible itineraries on the temporal multiplex. Each of these corresponds to M shortest paths on the static graph, because if a copy of v is connected to a copy of u, it is also connected to the copies of u at the same time-step on the other layers (through instantaneous switching links). Therefore, we estimate #shortest paths $= O(M)$. With this estimate, the second part of the algorithm has complexity $O(N^2M)$;

- In the third part, given all the shortest paths between the N^2 pairs of nodes, we compute the betweenness. This part has a complexity $O(N^3)$. In fact, we cannot apply efficient algorithms in the style of the Brandes algorithm [1], because such algorithm is based on the fact that subpaths of shortest paths are themselves shortest paths. This is not true in temporal networks. To understand this, consider the following example. The shortest path from A to D is a path departing at time t_1, passing from nodes B and C (3-legs itinerary), arriving at time t_2. However, the subpath from A to C is not the shortest path from A to C, because at a subsequent time t_3 there is a direct link from A to C. The latter cannot be part of the shortest path from A to D because after t_3 there are no links from C to D.

In conclusion, the overall complexity of the algorithm is typically dominated by the first part, with $O((NTM)^2 \log(NTM))$, except for networks with very large N, where the third phase might dominate with $O(N^3)$. In our empirical application, the first phase dominates.

References

[1] U. Brandes. A faster algorithm for betweenness centrality. *J. Math. Sociol.*, page 163–177, 2001.

[2] P. Grindrod, M. C. Parsons, D. J. Higham, and E. Estrada. Communicability across evolving networks. *Physical Review E*, 83(4):046120, 2011.

[3] Habiba, Chayant Tantipathananandh, and Tanya Berger-wolf. Betweenness Centrality Measure in Dynamic Networks. Technical report, DIMACS, 2007.

[4] H. Kim and R. Anderson. Temporal node centrality in complex networks. *Physical Review E*, 85:026107, 2012.

[5] D Taylor, SA Myers, A Clauset, MA Porter, and PJ Mucha. Eigenvector-based centrality measures for temporal networks. *Multiscale Model Simul.*, 15(1):537–574, 2017.

[6] Ioanna Tsalouchidou, Ricardo Baeza-yates, Francesco Bonchi, Kewen Liao, and Timos Sellis. Temporal betweenness centrality in dynamic graphs. *Int. J. Data Sci. Anal.*, 2019.

[7] S. Zaoli, P. Mazzarisi, and F. Lillo. Trip centrality: walking on a temporal multiplex with non-instantaneous link travel time. *Sci. Rep.*, 9(1):10570, 2019.
